# Performance of a Novel Low-Cost, Instrument-Free Plasma Separation Device for HIV Viral Load Quantification and Determination of Treatment Failure in People Living with HIV in Malaysia: a Diagnostic Accuracy Study

**DOI:** 10.1128/JCM.01683-18

**Published:** 2019-03-28

**Authors:** Minh D. Pham, Berhan A. Haile, Iskandar Azwa, Adeeba Kamarulzaman, Nishaan Raman, Alireza Saeidi, Maria Kahar Bador, Margaret Tan, Jiawei Zhu, Yi Feng, Julian H. Elliott, Mary L. Garcia, Fan Li, Suzanne M. Crowe, Stanley Luchters, David A. Anderson

**Affiliations:** a Burnet Institute, Melbourne, Australia; b Department of Epidemiology and Preventive Medicine, Monash University, Melbourne, Australia; c Infectious Disease Unit, Department of Medicine, Faculty of Medicine, University of Malaya, Kuala Lumpur, Malaysia; d Medical Microbiology Department, Faculty of Medicine, University of Malaya, Kuala Lumpur, Malaysia; e Nanjing BioPoint Diagnostics, Nanjing, People’s Republic of China; f Department of Population Health, Aga Khan University, Nairobi, Kenya; g International Centre for Reproductive Health, Department of Public Health and Primary Care, Ghent University, Ghent, Belgium; Memorial Sloan Kettering Cancer Center

**Keywords:** HIV, Malaysia, diagnostic accuracy, dried blood spot (DBS), plasma separation, viral load

## Abstract

HIV viral load (VL) testing is the recommended method for monitoring the response of people living with HIV and receiving antiretroviral therapy (ART). The availability of standard plasma VL testing in low- and middle-income countries (LMICs), and access to this testing, are limited by the need to use fresh plasma. Good specimen collection methods for HIV VL testing that are applicable to resource-constrained settings are needed. We assessed the diagnostic performance of the filtered dried plasma spot (FDPS), created using the newly developed, instrument-free VLPlasma device, in identifying treatment failure at a VL threshold of 1,000 copies/ml in fresh plasma. Performance was compared with that of the conventional dried blood spot (DBS). Venous blood samples from 201 people living with HIV and attending an infectious disease clinic in Malaysia were collected, and HIV VL was quantified using fresh plasma (the reference standard), FDPS, and DBS specimens. VL testing was done using the Roche Cobas AmpliPrep/Cobas TaqMan v2.0 assay. At a threshold of 1,000 copies/ml, the diagnostic performance of the FDPS was superior (sensitivity, 100% [95% confidence interval {CI}, 89.1 to 100%]; specificity, 100% [95% CI, 97.8 to 100%]) to that of the DBS (sensitivity, 100% [95% CI, 89.4 to 100%]; specificity, 36.8% [95% CI, 29.4 to 44.7%]) (*P* < 0.001). A stronger correlation was observed between the FDPS VL and the plasma VL (*r* = 0.94; *P* < 0.001) than between the DBS VL and the plasma VL (*r* = 0.85; *P* < 0.001). The mean difference in VL measures between the FDPS and plasma (plasma VL minus FDPS VL) was 0.127 log_10_ copies/ml (standard deviation [SD], 0.32), in contrast to –0.95 log_10_ copies/ml (SD, 0.84) between the DBS and plasma. HIV VL measurement using the FDPS outperformed that with the DBS in identifying treatment failure at a threshold of 1,000 copies/ml and compared well with the quantification of VL in plasma. The FDPS can be an attractive alternative to fresh plasma for improving access to HIV VL monitoring among people living with HIV on ART in LMICs.

## Abstract (precis)

HIV viral load (VL) testing is the recommended method for monitoring the response of people living with HIV and receiving antiretroviral therapy (ART). The availability of standard plasma VL testing in low- and middle-income countries (LMICs), and access to this testing, are limited by the need to use fresh plasma.

## INTRODUCTION

The World Health Organization recommends HIV-1 RNA viral load (VL) testing as the preferred method for monitoring the response to antiretroviral therapy (ART) in people living with HIV (1). Routine VL testing can support ART adherence (2) and enables early detection of treatment failure (3, 4). Existing evidence suggests that increasing the coverage of VL testing could (i) prolong sensitivity to first-line ART regimens, (ii) reduce the development of drug resistance in susceptible individuals, (iii) reduce the frequency of clinic visits required for disease monitoring in virally suppressed individuals, and (iv) increase the effectiveness and efficacy of treatment programs globally (5, 6). However, in many low- and middle-income countries (LMICs), access to and use of VL testing are still limited due to the complexity of specimen collection, storage, and transportation to central laboratories and the requirement for expensive laboratory equipment and highly trained personnel (7).

The dried blood spot (DBS) can be used as an alternative sample type to plasma for improving access to VL testing in LMICs (8). The use of DBS can simplify sample collection as well as transportation issues and can reduce the cost of VL testing by removing the need for cold-chain systems. DBSs can be stable for as long as 6 months at ambient temperature before testing (9). However, DBSs may have low sensitivity due to low sample volume (50 to 100 μl of whole blood) and low specificity in identifying treatment failure using the 1,000-copies/ml threshold due to the inclusion of white blood cells and associated viral RNA and proviral DNA (10). The reduced accuracy of the DBS for VL testing has limited its potential in expanding VL testing to all people living with HIV on ART in resource-constrained settings. Therefore, there is a need for technological innovation to improve the diagnostic accuracy of VL testing in resource-constrained settings.

A newly developed instrument-free, disposable, and easy-to-use sample collection and preparation device—the VLPlasma blood separation device—separates the plasma component from a whole-blood sample (100 µl) without the need for a centrifuge. This is a prototype product, fully manufactured by Nanjing BioPoint Diagnostics (Nanjing, People’s Republic of China) under ISO13485/2016 certification for study purposes. The device comprises a filter paper strip assembly that recovers cell-free plasma from whole blood using lateral flow principles, housed within a cartridge that protects the sample during drying and transport. Details of the development and evaluation of the lateral flow separation technique have been reported elsewhere (11). The plasma sample is dried and stored within the VLPlasma device cartridge, and the filter containing the dried plasma (known as the filtered dried plasma spot [FDPS]) is then transferred directly to a sample tube for VL testing, as an alternative to the DBS.

This study assessed the diagnostic accuracy of FDPSs, collected using the VLPlasma device, in the measurement of HIV-1 RNA VLs and the detection of VLs of ≤1,000 copies/ml, using fresh plasma specimens as the reference standard. We also compared the performance of FDPS with that of DBS for VL testing among people living with HIV and receiving ART at an infectious disease clinic in Kuala Lumpur, Malaysia.

## MATERIALS AND METHODS

This was a prospective diagnostic accuracy study. Each study participant provided 12 ml of venous blood collected in EDTA tubes for the preparation of fresh plasma, DBS, and FDPS samples. Three VL tests were carried out for each participant, and clinical decisions were based on the results of VL tests carried out on fresh plasma as part of the standard of care. HIV-1 RNA VLs were measured using the Roche Cobas AmpliPrep/Cobas TaqMan HIV-1 test, v2.0 (CAP/CTM), system, one of the most commonly used HIV VL testing platforms in low- and middle-income countries (12) with acceptable accuracy (13).

### Participants.

People aged 18 years and older living with HIV and attending the Infectious Disease Clinic, University Malaya Medical Centre, were consecutively recruited and invited to participate in the study. To be eligible, study participants had to have documented and confirmed HIV infection, be eligible for VL testing as part of the standard of care, and be currently taking ART at the study site with at least one of the following: (i) documented virological failure (defined as having two consecutive VL measurements exceeding 1,000 copies/ml) at previous visits during the past 6 months, (ii) documented immunological failure (defined as having a CD4 count below the baseline CD4 level or a persistent CD4 count below 100 cells/mm^3^), (iii) clinical failure (defined as having a new or recurrent clinical event indicating severe immunodeficiency, WHO clinical stage 4) in previous visits during the past 6 months, or (iv) documented self-reported nonadherence (defined as having missed ≥2 doses of medication) in the past 4 weeks. Participants also had to be able and willing to provide written informed consent.

### Sample size.

Based on our previous laboratory evaluation (11), the expected sensitivity (and specificity) of the FDPS in detecting samples with VLs at a cutoff of 1,000 copies/ml was at least 90%. Using formulae of sample size calculation for diagnostic test accuracy studies (14) with a marginal error (the difference between the estimates and the true value that is expected to be detected or one-half of the desired width of the confidence interval) of 0.05 and an estimated prevalence of treatment failure (VL, ≥1,000 copies/ml) at the study site of 20%, the required sample size was 173. With an expected response rate of at least 90%, the final sample size required for the study was 190.

### Plasma preparation.

Upon arrival at the laboratory (within 6 h of blood collection), 3 ml of EDTA–whole blood was centrifuged at 3,200 rpm for 20 min, and plasma aliquots were stored in sterile tubes at –4°C. The tubes were labeled with deidentified participant information for VL testing within 24 h of blood collection according to the standard operating procedure at the study site.

### FDPS preparation.

FDPS samples were prepared by a trained research laboratory staff member using a VLPlasma device (see Picture S1 in the supplemental material) in the following steps: (i) 100 µl of EDTA-blood was transferred to the sample cavity of the device using a calibrated pipette; (ii) after 3 min of incubation, 90 µl of phosphate-buffered saline (PBS) was added to the sample cavity by use of the plastic pipette and buffer provided with the VLPlasma device; (iii) the device was left to dry overnight at room temperature before being packaged into a ziplock bag with four desiccant sachets and a humidity indicator. FDPS samples were stored as long as 81 days at room temperature before testing.

### DBS preparation.

The same research staff member who prepared the FDPS sample also prepared the DBS sample. A 700-µl volume of EDTA-blood was spotted onto two Whatman no. 903 Protein Saver cards (Whatman plc, Little Chalfont, Buckinghamshire, UK) using a calibrated pipette, each containing five DBSs (70 µl for each DBS). The card was dried overnight and was then packaged and stored by the same procedure as that for the FDPS samples.

### HIV RNA VL quantification.

Plasma specimens were tested on the Roche CAP/CTM platform (Roche Diagnostics Ltd., Risch-Rotkreuz, Switzerland) at the viral diagnostic laboratory, University of Malaya, Malaysia, a center that participates in the College of American Pathologists quality assurance program. Plasma VL tests were carried out following the HI2CAP protocol developed by Roche Diagnostics for HIV VL testing using EDTA-plasma samples (15). The sample volume was 1.0 ml with a reportable VL range of 20 to 10,000,000 copies/ml as per the manufacturer’s instructions. Matched FDPS and DBS specimens were tested on the same Roche CAP/CTM platform using the HI2DFS protocol (Roche Diagnostics Ltd., Risch-Rotkreuz, Switzerland) specified for testing dried fluid samples and with a lower limit of quantification of 400 copies/ml. A single DBS cut from the card, or a plasma filter pad removed from the device, was placed in a sample tube (S-tube), to which was added 1 ml of guanidinium-based specimen pre-extraction reagent (SPEX; provided by Roche Diagnostics). The tube was then placed in a thermomixer (Eppendorf AG, Hamburg, Germany) for 10 min at 56°C for continuous shaking at 1,000 rpm. Nucleic acids were extracted using the automated Cobas AmpliPrep instrument and were then transferred to the Cobas TaqMan analyzer for amplification and quantification.

### Data management and analysis.

Data were collected using predeveloped data collection forms documenting participants’ age, gender, duration on ART, and eligibility status. HIV VL test results (FDPS, DBS, and fresh plasma) were recorded separately for each sample taken and tested. All data were entered and stored in the REDCap electronic data capture tool (Vanderbilt University, Nashville, TN, USA) hosted at the Burnet Institute (16).

The diagnostic performance of the FDPS was assessed by calculating sensitivity, specificity, positive predictive value (PPV), and negative predictive value (NPV) in identifying treatment failure at a threshold of 1,000 copies/ml—the current WHO-recommended virological failure threshold—in relation to the plasma reference standard for the VL. The diagnostic performance of the DBS was assessed identically to the performance of the FDPS. Kappa scores were used to assess diagnostic agreement between the index (FDPS or DBS) and the reference (plasma) method at a cutoff of 1,000 copies/ml.

For continuous outcomes (quantitative HIV-1 RNA VL measurements, reported as numbers of copies per milliliter), Pearson statistics and linear regression analyses were used to assess correlations between paired VL results (FDPS VL versus plasma VL and DBS VL versus plasma VL). Concordances (agreements) between FDPS and plasma VLs and between DBS and plasma VLs were assessed using Bland-Altman (BA) analysis (17) and Lin’s concordance correlation coefficient (p_c_) (18). The BA analysis evaluates the mean difference between measurements with the index method and measurements with the reference method (calculated as plasma VL minus FDPS VL or plasma VL minus DBS VL) and estimates the limits of agreement (LOAs), within which 95% of the differences fall. The closer the mean difference is to zero (perfect agreement) and the narrower the width of the 95% LOAs, the better the agreement between the two methods. The p_c_ quantifies the agreement between the two measures of the same variable (VL); p_c_ values range from −1 to 1, with perfect agreement at 1.

A volume correction factor of 1.97 was applied for quantitative FDPS VLs (>400 copies/µl). This correction factor was the average of the ratio of the plasma VLs to the corresponding quantitative FDPS VLs (*n* = 32). As per the manufacturer’s package insert instructions (19), no correction factor was applied for DBS VLs on the Roche CAP/CTM platform.

All analyses were performed using STATA statistical software (version 15.0; StataCorp, College Station, TX). Significance levels were set at an α value of <0.05.

### Ethics.

This study received ethics approval from the Medical Research Ethics Committee of the University of Malaya, Kuala Lumpur, Malaysia (no. 20161021-4399) and the Ethics Committee of The Alfred Hospital, Melbourne, Australia (no. 478/16).

## RESULTS

**FIG 1:**
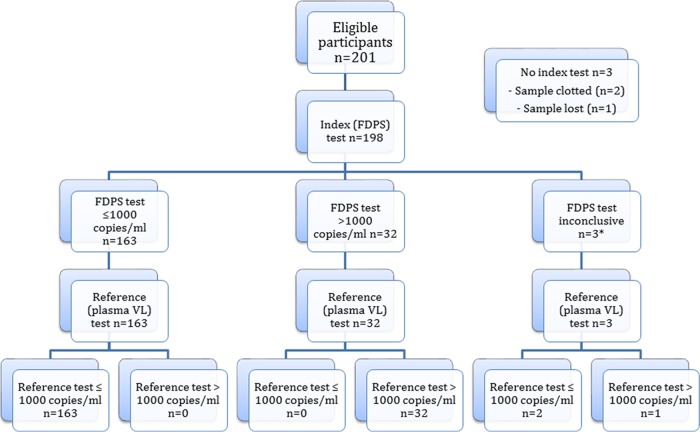
Flow diagram of FDPS VL test performance at a cutoff of 1,000 copies/ml according to STARD guidelines. The asterisk indicates that three FDPS samples failed to provide a valid viral load result on the Roche Cobas AmpliPrep/Cobas TaqMan platform.

**FIG 2:**
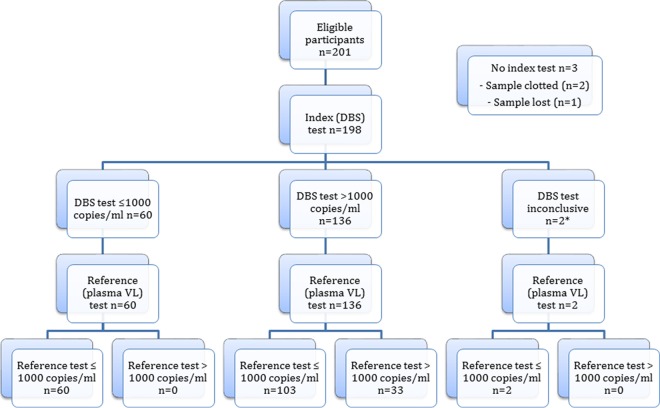
Flow diagram of DBS VL test performance at a cutoff of 1,000 copies/ml according to STARD guidelines. The asterisk indicates that two DBS samples failed to provide a valid viral load result on the Roche Cobas AmpliPrep/Cobas TaqMan platform.

From June 2017 to May 2018, a total of 201 people living with HIV on ART who met the inclusion criteria and provided written consent were enrolled into the study. Three patients did not have index VL tests (FDPS and DBS) done (Fig. 1 and 2). (Figures 1 and 2 are STARD [Standard for Reporting of Diagnosis Accuracy Studies] flow diagrams [20].) The mean age of the remaining 198 patients was 41.7 years (standard deviation [SD], 10.3 years; range, 21 to 69 years), with a median duration on ART of 36 months (interquartile range [IQR], 12 to 84 months). Among 198 participants who had three VL tests each (plasma VL, FDPS VL, DBS VL) done, 3 did not have valid FDPS VL results and 2 did not have valid DBS VL results. This left 195 participants with paired plasma VL–FDPS VL results and 196 participants with paired plasma VL–DBS VL results for analysis (Fig. 1 and 2; see also Table S1 in the supplemental material). Reported plasma VLs ranged from ND (VL not detected) to 1,593,975 copies/ml, while the reported FDPS VLs ranged from ND to 1,530,634 copies/ml, and the reported DBS VLs ranged from ND to 1,440,221 copies/ml.

### Diagnostic accuracy of the FDPS for VL assessment.

At a cutoff of 1,000 copies/ml, agreement between FDPS VLs and plasma VLs was 100% (*n* = 195) (Fig. 1). Sensitivity, specificity, positive predictive value (PPV), and negative predictive value (NPV) in identifying treatment failure using FDPS were 100% (95% confidence interval [CI], 89.1% to 100%), 100% (95% CI, 97.8% to 100%), 100% (95% CI, 89.1% to 100%), and 100% (95% CI, 97.8% to 100%), respectively. The kappa score for the measurement of agreement between FDPS VLs and plasma VLs was 1.000 (95% CI, 1.000 to 1.000).

**FIG 3:**
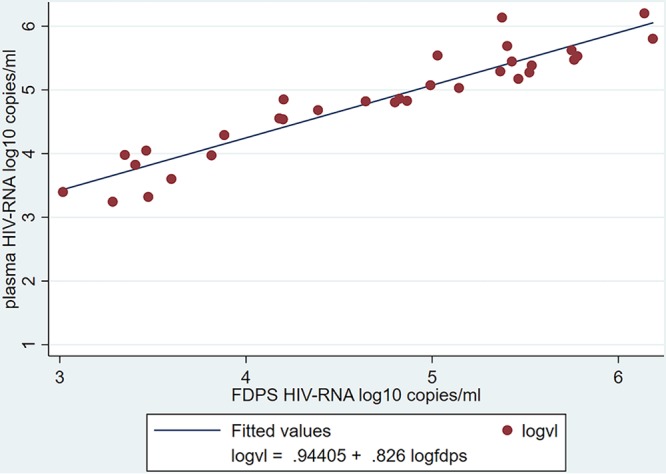
Correlation between paired VL results obtained from FDPS and plasma specimens, measured in log_10_ copies per milliliter (*n* = 32). Pearson correlation coefficient (*r*), 0.94; *P* < 0.001.

**FIG 4:**
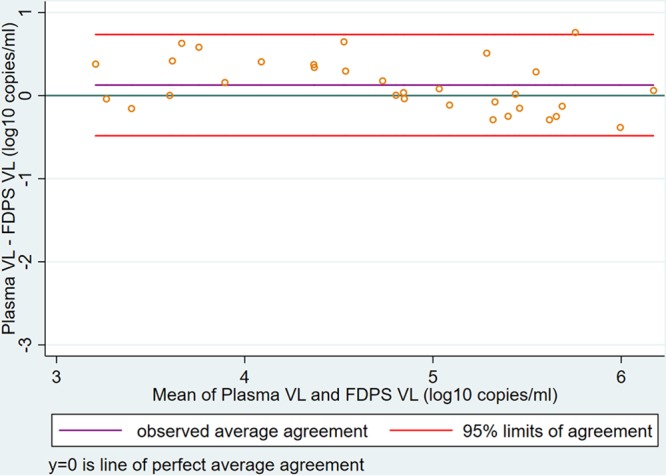
Bland-Altman plot demonstrating agreement between FPDS and plasma VL results, measured in log_10_ copies per milliliter (*n* = 32). The mean difference (purple line) was 0.127 (SD, 0.31) log_10_ copies/ml, and the 95% limits of agreement (red lines) were –0.48 to 0.74 log_10_ copies/ml.

In the comparison of diagnostic agreement between FDPS VLs and plasma VLs as continuous measurement, we found that of 198 participants, 32 had quantifiable VL results on both the FDPS and the plasma samples (plasma VL, >20 copies/ml; FDPS VL, >400 copies/ml). Pearson correlation and linear regression showed a strong, positive correlation between FDPS VLs and plasma VLs (*r* = 0.94; *P* < 0.001; *n* = 32) (Fig. 3). BA analysis showed a positive mean difference of 0.127 (SD, 0.31) log_10_ copies/ml between FDPS and plasma VL results and 95% limits of agreement (LoAs) of −0.48 to 0.74 log_10_ copies/ml (Fig. 4). Lin’s concordance correlation coefficient for the FPDS was 0.927.

### Diagnostic accuracy of the DBS for VL assessment.

At a cutoff of 1,000 copies/ml, the agreement between DBS VLs and plasma VLs was 47% (95% CI, 40.5% to 54.4%) (*n* = 196) (Fig. 2). The sensitivity, specificity, PPV, and NPV of the DBS were 100% (95% CI, 89.4% to 100%), 36.8% (95% CI, 29.4% to 44.7%), 24.3% (95% CI, 17.3% to 32.4%), and 100% (95% CI, 94.0% to 100%), respectively. The kappa score for the measurement of agreement between DBS VLs and plasma VLs was 0.164 (95% CI, 0.103 to 0.225).

**FIG 5:**
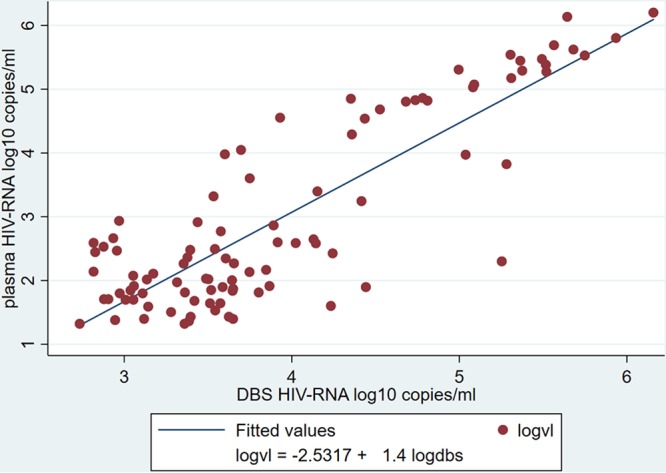
Correlation between paired VL results obtained from DBS and plasma samples, measured in log_10_ copies per milliliter (*n* = 95). Pearson correlation coefficient (*r*), 0.85; *P* < 0.001.

**FIG 6:**
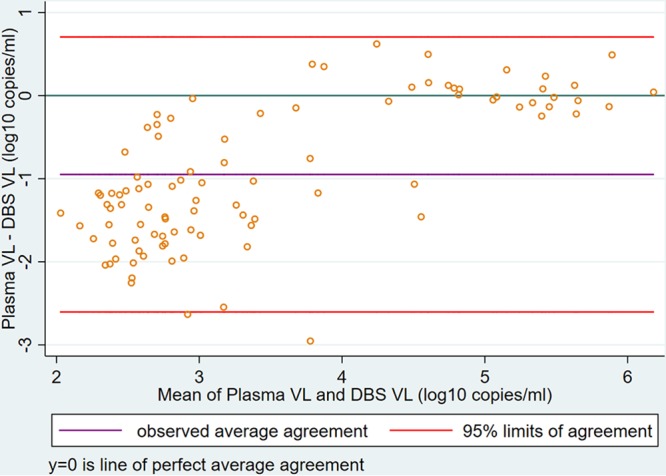
Bland-Altman plot demonstrating agreement between DBS and plasma VL results, measured in log_10_ copies per milliliter (*n* = 95). The mean difference (purple line) was –0.95 (SD, 0.84) log_10_ copies/ml, and the LoAs (red lines) were –2.60 to 0.71 log_10_ copies/ml.

For the comparison of continuous measurements, 95 patients had quantitative VL results with paired DBS–plasma samples. Pearson correlation and linear regression analyses showed a positive correlation (*r* = 0.85; *P* < 0.001; *n* = 95) (Fig. 5). BA analysis showed an overestimation of VLs for the DBS (relative to plasma), with a mean difference of −0.95 (SD, 0.84) log_10_ copies/ml and LoAs of −2.60 to 0.71 log_10_ copies/ml (Fig. 6). Lin’s concordance correlation coefficient for the DBS was 0.581.

Detailed data on the diagnostic performance of the FDPS and DBS are presented in Tables S1 to S4 in the supplemental material.

## DISCUSSION

In this study, we evaluated the performance of a novel plasma separation device and method providing a filtered dried plasma sample to allow easy transport of a plasma sample to the laboratory for HIV VL testing. Using a virological failure threshold of 1,000 copies/ml, the current WHO-recommended cutoff for diagnosis of treatment failure, we observed perfect (100%) agreement between FDPS and plasma results. We found that the FDPS outperforms the DBS in identifying virological failure among people living with HIV on ART by use of the Roche CAP/CTM system. The DBS misclassified 53% (103/196) of patients as “failing treatment,” requiring unnecessarily close monitoring, additional adherence consultation, confirmatory VL testing, and switching of treatments. This finding is in line with findings from prior studies of the DBS for HIV VL testing. A systematic review of 43 studies comparing DBS and plasma specimens for VL testing, based on which WHO recommended the use of the DBS for VL monitoring at a threshold of 1,000 copies/ml, reported pooled estimates of sensitivity and specificity for the Roche CAP/CTM assay (SPEX protocol) of 99% (95% CI, 97 to 100%) and 44% (95% CI, 18 to 74%), respectively. When used in different assays/protocols (Roche CAP/CTM FVE protocol, Abbott RealTime assay, bioMérieux NucliSens test, Biocentric Charge Virale, Siemens kPCR), the DBS produces higher specificity (range, 55 to 95%) but lower sensitivity (range, 84 to 95%) (8).

Quantitatively, while strong and positive correlation with plasma was found for both the FDPS and the DBS by Pearson’s correlation analysis, Bland-Atman analysis results showed that FDPS had much better agreement with a much smaller bias (0.13 versus −0.95 log copies/ml), and a narrower limit of agreement (−0.48 to 0.74 versus −2.60 to 0.71 log copies/ml); furthermore, all samples were within the limit of agreement. Better performance of the FDPS was confirmed by Lin’s concordance analysis, with a concordance coefficient (p_c_) of 0.93 versus 0.58 for the DBS (a p_c_ value of 1 is a perfect concordance).

We observed an overall underestimation of HIV-RNA VLs with the FDPS relative to levels for corresponding plasma specimens. This could be explained by the differences in the volume of specimen used. The FDPS used 100 μl of whole blood, while HIV RNA was measured in 1 ml of the fresh plasma sample. Even though the Roche CAP/CTM system has incorporated a correction in the HI2DFS testing protocol for dried fluid samples, this could still be a source of bias for HIV RNA quantification with FDPS samples. To compensate for the volume difference, for samples with quantitative FDPS VL results (>400 copies/ml), we applied a correction factor of 1.97. This was calculated on the basis of results of only 32 paired FDPS–plasma samples with quantitative VLs on the Roche CAP/CTM system. Given different sample handling/processing procedures, as well as different techniques in HIV RNA amplification and quantification across commercially available VL testing platforms, this correction factor is likely to be altered in future studies validating the performance of the FDPS on different VL platforms and with a larger sample size.

There are some limitations that should be taken into consideration in interpreting the findings of this study. First, in our study sample, the treatment failure rate using a threshold of 1,000 copies/ml was 17%, with plasma VLs ranging from undetectable to 6.2 log_10_ copies/ml. While this failure rate falls within the range of virological failure rates of people living with HIV on ART reported from other studies in resource-constrained settings, the virological failure rate in LMICs could be as high as 30% for patients at higher levels of viremia (21, 22). We were unable to assess the performance of the FDPS in samples with plasma VLs of >6 log_10_ copies/ml—a common scenario among ART-naïve patients who present late in care. Second, we had only 32 paired FDPS–plasma samples with quantitative VL results, which was about the minimum sample size required for an assessment of agreement between two methods of measurement using Bland-Altman analysis (23). We were unable to perform subanalyses of these samples to provide further insights into the performance of the FDPS compared to plasma quantitatively (continuous measurement of HIV VL). Third, in this study, we used venous blood samples collected by a trained clinical staff. Since venipuncture may not be feasible and/or practical at clinics in resource-constrained settings, and there are potential differences between the diagnostic performance of dried fluid samples prepared from venous blood and those prepared from capillary blood (24), further study is needed to assess the performance of the FDPS using finger pricks by health care professionals at the point of care.

In conclusion, we observed excellent diagnostic accuracy for the FDPS, in the hands of highly trained clinical and laboratory staff, in detecting treatment failure at a cutoff of 1,000 copies/ml. There were a strong correlation and excellent agreement between FDPS and plasma VL results obtained from venous blood samples on the Roche CAP/CTM platform. This is in stark contrast to the low specificity observed with the DBS. Our study findings show that the FDPS could potentially be an alternative specimen to fresh plasma for HIV VL monitoring in LMICs. Further study assessing the diagnostic performance of the FDPS, using capillary blood samples in a clinic setting with a larger sample size, is warranted.

## Supplementary Material

Supplemental file 1
