# Supplementary material for: Performance of a Novel Low-Cost, Instrument-Free Plasma Separation Device for HIV Viral Load Quantification and Determination of Treatment Failure in People Living with HIV in Malaysia: a Diagnostic Accuracy Study
Source: J Clin Microbiol. 2019 Mar 28;57(4):e01683-18. doi: 10.1128/JCM.01683-18 (PMC6440787; doi:10.1128/JCM.01683-18)
Supplement: Supplemental file 1 [file JCM.01683-18-s0001.pdf]

Supplementary data file

Picture 1. Schematic and functionality of the VLPlasma device

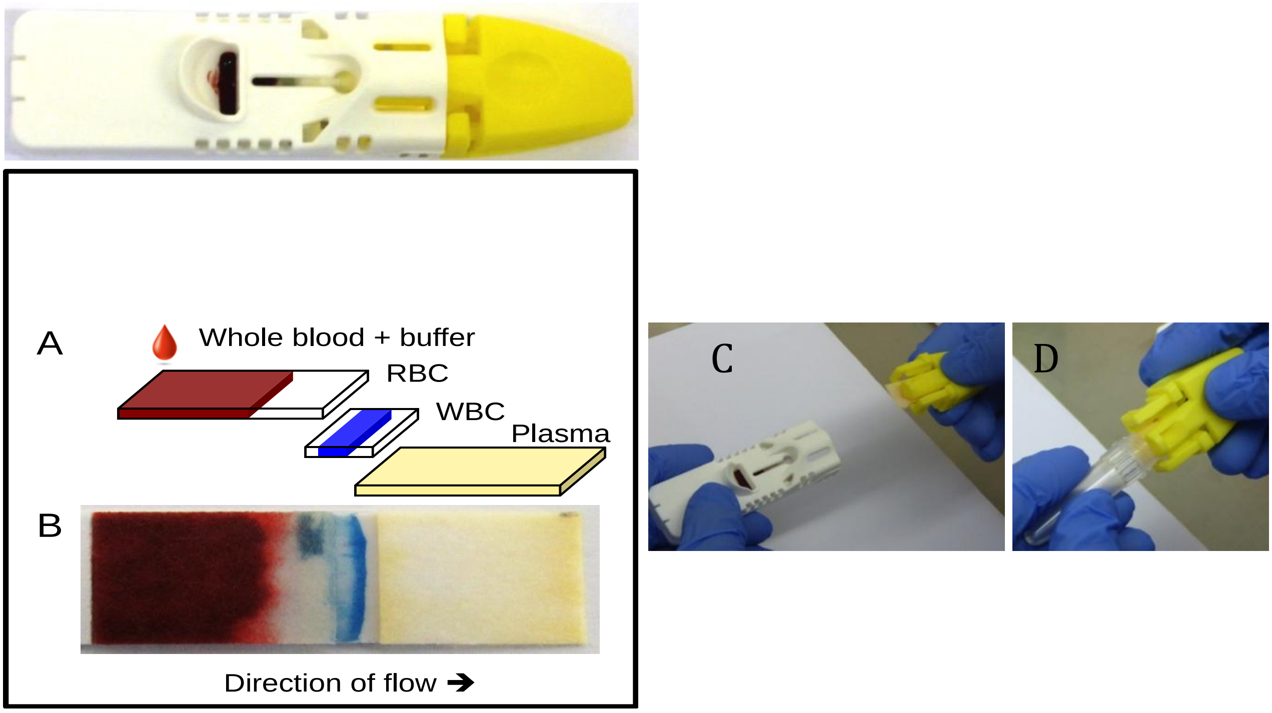

The FDPS (VL-Plasma Blood Separation Device) utilises patented technology to retain red blood cells (RBC) by immune aggregation and white blood cells (WBC) by membrane filtration (shown schematically in A); and in an isolated separation strip (shown schematically in B), where 10 μm blue beads were added to visualise the retained WBC. Near quantitative (94%) recovery of plasma is achieved in the plasma strip (Whatman 903). The plasma strip is designed to be detached (safe removal of plasma pad held in yellow “gripper” shown in C) and the plasma pad is placed into the sample tube (shown in D) for VL testing.

Table 1: Sensitivity, specificity, positive predictive value/PPV, negative predictive value/NPV (95% confidence intervals) of FDPS and DBS at 1,000 copies/ml threshold, and at the limit of quantification (400 copies/ml) for HIV viral load testing compared to fresh plasma on the Roche CAP/CTM (B).

Performance at 1000 copies/mL cut-off					
		FDPS VL (copies/ml)			
		>1,000	≤1,000	Total	
Plasma	>1,000	32	0	32	Sensitivity: 100% (89.1%-100%)<br>Specificity: 100% (97.8%-100%)
	≤1,000	0	163	163	PPV: 100% (89.1%-100%)<br>NPV: 100% (97.8%-100%)
	Total	32	163	195	
		DBS VL (copies/ml)			
		>1,000	≤1,000	Total	
Plasma	>1,000	33	0	33	Sensitivity: 100% (89.4%-100%)<br>Specificity: 36.8% (29.4-44.7%)<br>PPV: 24.3% (17.3%-32.4%)<br>NPV: 100% (94.0%-100%)
	≤1,000	103	60	163	
	Total	136	60	196	
Performance at 400 copies/mL cut-off					
		FDPS VL (copies/ml)			
		>400	≤400	Total	
Plasma	>400	32	6	38	Sensitivity: 84.2% (68.7%-94.0%)<br>Specificity: 100% (97.7%-100%)<br>PPV: 100% (89.1%-100%)<br>NPV: 96.3% (92.2%-98.6%)
	≤400	0	157	157	
	Total	32	163	195	
		DBS VL (copies/ml)			
		>400	≤400	Total	
Plasma	>400	39	0	39	Sensitivity: 100% (91.0%-100%)<br>Specificity: 14% (9.0%-20.4%)<br>PPV: 22.4% (16.5%-29.3%)<br>NPV: 100% (84.6%-100%)
	≤400	135	22	157	
	Total	174	22	196	

PPV: Positive Predictive Value; NPV: Negative Predictive Value

FDPS= Filtered Dried Plasma Spot; DBS=Dried Blood Spot

Table 2: Detection and agreement rates of filtered dry plasma spot (FDPS) by corresponding plasma VL results from paired FDPS and plasma samples of people living with HIV attending infectious disease unit, UoM Medical Centre, Malaysia (N=195)

Plasma VL (copies/ml)	FDPS VL (copies/ml)						Number of paired FDPS & Plasma VL results	Proportion of FDPS samples tested with detectable VL>400 copies/ml, n/N (%)	Agreement between paired DBS & plasma VL, n/N (%)
	ND	<400	400 - 1,000	1,000-10,000	10,000-100,000	>100,000			
ND	55	2	0	0	0	0	57	0/57 (0)	57/57 (100)
<20	39	3	0	0	0	0	42	0/42 (0)	42/42 (100)
20-400	40	18	0	0	0	0	58	0/58 (0)	58/58 (100)
400-1,000	3	3	0	0	0	0	6	0/6 (0)	0/6 (0.0)
1,000-10,000	0	0	0	7	0	0	7	7/7 (100)	7/7 (100)
10,000-100,000	0	0	0	2	8	0	10	10/10 (100)	8/10 (80)
>100,000	0	0	0	0	1	14	15	15/15 (100)	14/15 (93)
Total	137	26	0	9	9	14	195	32/195 (16)	186/195 (95)

ND=Not Detected

Table 3: Detection and agreement rates of dried blood spot (DBS) by corresponding plasma VL results from paired DBS and plasma samples of people living with HIV attending infectious disease unit, UoM Medical Centre, Malaysia (N=196)

Plasma VL (copies/ml)	DBS VL (copies/ml)						Number of paired DBS & Plasma VL results	Proportion of DBS samples tested with detectable VL>400copies/ml, n/N (%)	Agreement between paired DBS & plasma VL, n/N (%)
	ND	<400	400-1,000	1,000-10,000	10,000-100,000	>100,000			
ND	1	12	16	26	1	0	56	43/56 (76.8)	13/56 (23.2)
<20	2	4	10	26	0	0	42	36/42 (85.7)	6/42 (14.3)
20-400	1	2	10	40	5	1	59	56/59 (94.9)	3/59 (5.1)
400-1,000	0	0	2	3	1	0	6	6/6 (100)	2/6 (33.3)
1,000-10,000	0	0	0	3	2	2	7	7/7 (100)	3/7 (42.9)
10,000-100,000	0	0	0	2	8	0	10	10/10 (100)	8/10 (80.0)
>100,000	0	0	0	0	1	15	16	16/16 (100)	15/16 (93.8)
Total	4	18	38	100	18	18	196	174/196 (88.8)	50/196 (25.5)

ND=Not Detected

Table 4: Discordant VL results among six patients with low-level viremia (400 to 1000 copies/ml) in plasma

No	Plasma VL (copies/ml)	DBS VL (copies/ml)	FDPS VL (copies/ml)
1.	443	13,485	ND
2.	461	860	ND
3.	591	3,770	<400
4.	732	7,788	ND
5.	823	2,741	<400
6.	863	932	<400
